# A 27-Year-Old Patient Fulfilling the Diagnostic Criteria of Both CMML and JMML

**DOI:** 10.1155/2016/7543582

**Published:** 2016-02-29

**Authors:** Assem A. Elghazaly, Mohmmed U. Manzoor, Mai A. AlMishari, Mamoun H. Ibrahim

**Affiliations:** ^1^Department of Adult Hematology/Cancer, King Fahad Medical City, Al Dabab Street, Al Sulaimaniyah, P.O. Box 59046, Riyadh 11525, Saudi Arabia; ^2^Department of Medical Imaging, King Fahad Medical City, Al Dabab Street, Al Sulaimaniyah, P.O. Box 59046, Riyadh 11525, Saudi Arabia; ^3^Department of Clinical Laboratory, King Fahad Medical City, Al Dabab Street, Al Sulaimaniyah, P.O. Box 59046, Riyadh 11525, Saudi Arabia

## Abstract

Chronic myelomonocytic leukaemia (CMML) and juvenile myelomonocytic leukaemia (JMML) are two disease entities that come under the myelodysplastic/myeloproliferative neoplasms category. Each of the two conditions has its own diagnostic criteria. In addition, they have different ages of presentation; while CMML is typically a disease of the elderly, JMML is a disease of young children. Here we are presenting the case of a 27-year-old male patient who, at the time of diagnosis, fulfilled the diagnostic criteria of both diseases. In addition he had radiological changes of type 1 neurofibromatosis. Possible explanations of the patient case have been discussed.

## 1. Introduction

CMML and JMML are clonal hematological malignancies, classified by the WHO as myelodysplastic/myeloproliferative neoplasms [1]. CMML is a rare disorder with estimated incidence of <1 case per 100 000 persons per year [2]. The median age at presentation is 65–75 years. In addition to persistent peripheral blood monocytosis, presenting manifestations may also include those of bone marrow failure, general symptoms, splenomegaly, and hepatomegaly. The WHO diagnostic criteria for CMML are shown as follows [3].

*Diagnostic Criteria for Chronic Myelomonocytic Leukaemia*

1. Persistent peripheral blood monocytosis >1 × 10^9^/L.
2. No Philadelphia chromosome or BCR-ABL1 fusion gene.
3. No arrangement of PDGFRA or PDGFRB (which should be specifically excluded in cases with eosinophilia).
4. Fewer than 20% blasts in the peripheral blood and the BM (blasts including myeloblasts, monoblasts, and promonocytes).
5. Dysplasia in one or more cell lines. If dysplasia is absent or minimal, the diagnosis of CMML may still be made if the other requirements are met and
  - an acquired clonal cytogenetic abnormality or molecular genetic abnormality present in hematopoietic cells or
  - the monocytosis has persisted for at least 3 months
  - all other causes of monocytosis have been excluded.

An overall median survival for CMML patients is about 12–24 months [4]. Effective therapy is limited, with allogeneic stem cell transplantation being the only known curative regimen for CMML [5].

The median age at presentation of JMML is 2 years (range 0.1–11.4) [6]. JMML is reported to have an incidence of 1.2 per million child per year [7]. At the molecular level, 35% of patients have gain-of-function mutations in* PTPN11* and 35% gain-of-function mutations in* NRAS* or* KRAS* (*RAS pathway signals*) [8]. An association does exist between JMML and NF1 gene suppression. About 30% of patients with JMML have NF1 gene inactivation, while only 10% to 14% of children with JMML have a clinical diagnosis of neurofibromatosis, type 1. Young children with NF1 have a 200- to 500-fold increase in the risk of developing malignant myeloid disorders, particularly JMML, whereas adults with NF1 do not show an increased susceptibility to leukaemia [8, 9].

Presenting manifestations of JMML may include, in addition to monocytosis in the peripheral blood, fever, pallor, skin rash, hepatosplenomegaly, and lymphadenopathy. In the vast majority of cases, JMML is an aggressive and fatal disorder if left untreated; the median survival time of children who do not receive an allograft can be as short as 10 to 12 months [6]. The currently used JMML diagnostic criteria are shown as follows [10, 11].

*JMML Diagnostic Criteria*. Suggestive clinical features are as follows:

 Hepatosplenomegaly.
 Lymphadenopathy.
 Pallor.
 Fever.
 Skin rash.

Laboratory criteria (all three must be met) are as follows:

1. Persistent peripheral blood monocytosis (>1 × 10^9^/L).
2. No Philadelphia chromosome or BCR-ABL fusion gene.
3. <20% myeloblasts or monoblasts in the marrow.

Further criteria to be met (need to fulfill at least two) are as follows:

1. Increased hemoglobin F (corrected for age).
2. Immature myeloid precursors on the peripheral blood smear.
3. Peripheral blood white blood cell count >10 × 10^9^/L.
4. Clonal cytogenetic abnormalities (including monosomy 7).
5. GM-CSF hypersensitivity of myeloid progenitors (in vitro test).

More recently updated clinical and laboratory diagnostic criteria for JMML have been proposed that incorporate* NF1*,* RAS*, and* PTPN11* mutational status into the diagnostic assessment as follows [8].

*Updated Clinical and Laboratory Diagnostic Criteria of JMML*

*Category 1*. All of the Following

 Splenomegaly.
 Absolute monocyte count > 1000/*μ*L.
 Blasts in PB/BM <20%.
 Absence of the t(9;22) BCR/ABL fusion gene.
 Age less than 13 years.

*Category 2*. At least 1 of the following

 Somatic mutation in* RAS* or* PTPN11*.
 Clinical diagnosis of NF1 or* NF1* gene mutation.
 Monosomy 7.

*Category 3*. At least 2 of the following

 Circulating myeloid precursors.
 WBC > 10,000/*μ*L.
 Increased fetal hemoglobin (HgF) for age.
 Clonal cytogenetic abnormality excluding monosomy 7.

## 2. Case History

A 27-year-old male patient with an unremarkable medical history presented with a 2-month history of fatigue, loss of weight, early satiety, and abdominal discomfort. There was no significant family history. On clinical examination he was found to have massive splenomegaly, with the lower pole reaching the right iliac fossa.

**Figure 1:**
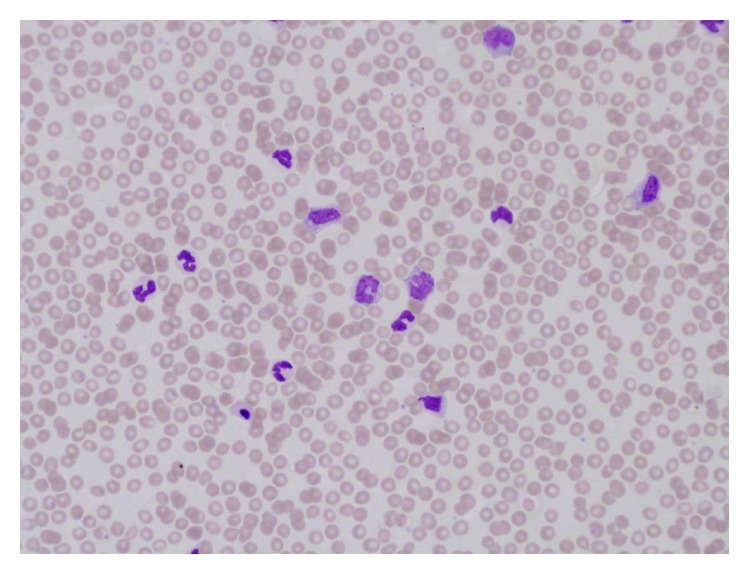
Peripheral blood film showing left shift and monocytes.

**Figure 2:**
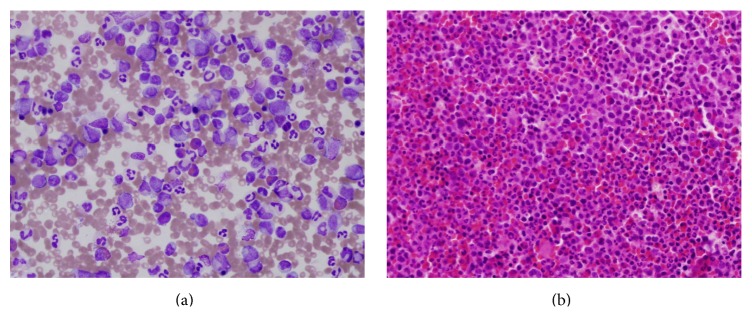
(a) Bone marrow aspiration and (b) bone marrow biopsy show increased cellularity, decreased erythroid precursors, and increased myeloid precursors with progressive and orderly maturation.

The blood count showed WBC 85.4 × 10^9^/L, neutrophil 49.5 × 10^9^/L, lymphocytes 5.1 × 10^9^/L, monocytes 13.6 × 10^9^/L, eosinophils 2.5 × 10^9^/L, basophils 0.8 × 10^9^/L, hemoglobin 12.6 g/dL, and platelets 81 × 10^9^/L. Peripheral blood morphology revealed left shift with 1-2% myeloid blasts (Figure 1). Examination of the bone marrow aspiration and biopsy showed cellularity of 100%, adequate megakaryocytes, decreased erythroid precursors, and increased myeloid precursors with progressive and orderly maturation. No lymphoid aggregates or granulomas were seen (Figures 2(a) and 2(b)). A differential count performed on the bone marrow aspiration reported the following: myeloid of 81%, blasts 4%, erythroid 6% (pronormoblasts, basophilic, orthochromatic, and polychromatic normoblasts), lymphocytes 3%, and monocytes 6%.

Bone marrow FISH analysis in 200 nuclei showed no evidence of BCR/ABL1 t(9:22)(q34;q11.2) rearrangement, deletion 5q, or deletion 7q. BCR/ABL fusion gene by PCR was negative. Bone marrow chromosomal analysis by banding technique revealed 46XY, with no apparent numerical or structural abnormalities. Further FISH analysis showed no evidence of rearrangement of 5q33 (PDGFRB) or 4q12 (FIP1L1-PDGFRA). Unfortunately no molecular study for* RAS pathway* or* PTPN11* mutations was done.

**Figure 3:**
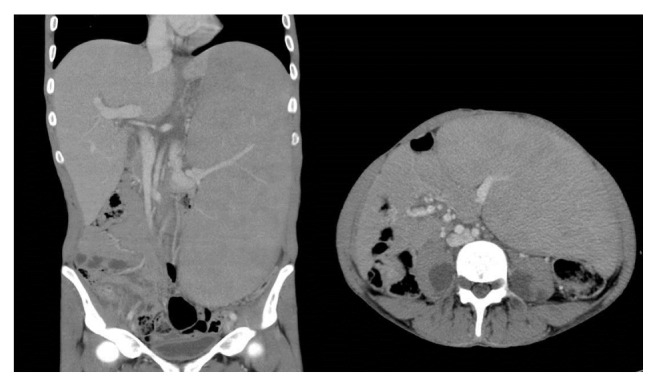
CT scan of the abdomen showing massive splenomegaly and bilateral plexiform neurofibromas along the psoas muscles.

**Figure 4:**
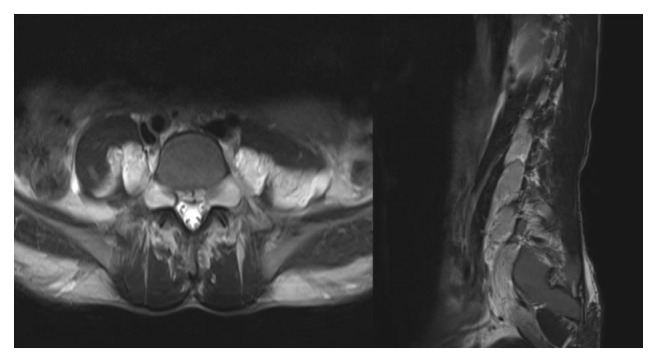
T2W MRI scan of the lumbar spine showing bilateral enlarged exit neural foramen along with extensive neurofibromas and bilateral plexiform neurofibromas.

A contrast enhanced CT scan of the neck, chest, abdomen, and pelvis was performed as a part of the initial workup which showed markedly enlarged spleen, reaching the pelvis and crossing the midline with no suspicious focal lesion. The liver was also mildly enlarged with no suspicious focal lesion. A few borderline enlarged cervical lymph nodes were present in the neck; however, no other significant lymphadenopathy was present in the chest, abdomen, or pelvis. Other than massive splenomegaly and mild hepatomegaly, CT showed symmetrical paraspinal hypodense masses with mildly enlarging neural foramina along the lumbar spine extending into the psoas muscle and along the course of sciatic nerves representing bilateral neurofibromas as well as plexiform neurofibromas, characteristic of neurofibromatosis type I (Figure 3). An MRI scan of the spine was performed, which confirmed the presence of bilateral neurofibromas along the cervical and lumbar spine, as well as bilateral plexiform neurofibromas (Figure 4). There was no evidence of dural ectasia, scoliosis, or any intramedullary abnormal signal. Unfortunately MRI of the brain was not performed in this patient to look for intracranial findings of neurofibromatosis type I (NF1). However, a CT brain was performed, which was unremarkable.

The neurofibromatosis type I testing via NF1 gene sequencing came as negative.

At this stage, the patient was having the diagnostic criteria of both CMML and JMML [3, 10, 11]. Considering the unfavorable nature of both diseases, the treatment plan was for the patient to receive an allogeneic stem cells transplantation (Allo-SCT). The patient received hydroxyurea for cytoreduction with a good response. After 4 months of HU therapy, the patient appreciated marked improvement of his general and abdominal symptoms with marked reduction of the splenic size. There was also improvement in the blood parameters: WBC 7.2 × 10^9^/L, neutrophils 2.6 × 10^9^/L, monocytes 1.9 × 10^9^/L, hemoglobin 13.5 g/dL, and platelets 84 × 10^9^/L. Five months from the diagnosis, the patient underwent Allo-SCT, from his HLA-identical brother.

## 3. Discussion

We present here a case of our patient who fulfilled all the WHO 2008 diagnostic criteria of CMML (see Section 1), namely, persistent peripheral blood monocytosis 1.9–13.6 × 10^9^/L for more than 4 months, no Philadelphia chromosome or* BCR-ABL1* fusion gene by FISH, chromosomal analysis by banding technique or PCR, and no rearrangement of* PDGFRA* or* PDGFRB*, with 4% blasts (including promonocytes) of the BM and with all other causes of monocytosis being excluded.

The patient age at the time of diagnosis was 27 years, which is clearly younger than the known median age of onset of 65–75 years for CMML. However, in two recent large studies, the age ranges of disease presentation were 20–93 years from the Mayo Clinic study [12] and 40–91 years for the Groupe Francophone des Myelodysplasies (GFM) study [13].

At the same time the patient demonstrated the currently used diagnostic criteria for JMML as well (see Section 1), that is, hepatosplenomegaly, persistent peripheral blood monocyte count > 1 × 10^9^/L, no Philadelphia chromosome or BCR-ABL fusion gene, bone marrow blasts 4%, myeloid blasts in the peripheral blood 1-2%, and peripheral blood white blood cell count 85.4 × 10^9^/L. However, our patient is obviously older than the reported age of onset of JMML, 0.1–11.4 years, as well as the proposed age of 13 years [8]. Ortiz et al. reported JMML in a 16-year-old patient with Noonan syndrome [14].

In spite of the clear radiological findings of bilateral lumber plexiform neurofibroma, cervical and lumber neurofibroma, and enlargement of the CSF spaces along the exit neural foramina bilaterally throughout the axial skeleton, it was not possible to put the diagnosis of NF1 as the other diagnostic criteria were not fulfilled [15].

Our young adult patient fulfilled the diagnostic criteria of both CMML and JMML and in addition had radiological changes of NF1. Such case, to our knowledge, has not been reported in the literature. It is possible that this patient had undelaying NF1 (with the acknowledged diagnostic limitations) which was complicated by JMML. Alternatively he might have got CMML at unusually younger age, with the accidently discovered radiological changes of NF1.

Future studies are needed to investigate whether NF1 has any association with CMML or not.
